# Bioactive Scaffolds Integrated with Liposomal or Extracellular Vesicles for Bone Regeneration

**DOI:** 10.3390/bioengineering8100137

**Published:** 2021-10-01

**Authors:** Minjee Kang, Chung-Sung Lee, Min Lee

**Affiliations:** 1 Division of Advanced Prosthodontics, School of Dentistry, University of California, Los Angeles, CA 90095, USA; mkang514@ucla.edu; 2 Department of Pharmaceutical Engineering and Biotechnology, Sun Moon University, Asan 31460, Korea; chungsunglee@sunmoon.ac.kr; 3 Department of Bioengineering, University of California, Los Angeles, CA 90095, USA

**Keywords:** liposome, exosome, extracellular vesicle, scaffold, drug delivery system, bone regeneration, bone tissue engineering

## Abstract

With population aging and increased life expectancy, an increasing number of people are facing musculoskeletal health problems that necessitate therapeutic intervention at defect sites. Bone tissue engineering (BTE) has become a promising approach for bone graft substitutes as traditional treatments using autografts or allografts involve clinical complications. Significant advancements have been made in developing ideal BTE scaffolds that can integrate bioactive molecules promoting robust bone repair. Herein, we review bioactive scaffolds tuned for local bone regenerative therapy, particularly through integrating synthetic liposomal vesicles or extracellular vesicles to the scaffolds. Liposomes offer an excellent drug delivery system providing sustained release of the loaded bioactive molecules. Extracellular vesicles, with their inherent capacity to carry bioactive molecules, are emerging as an advanced substitute of synthetic nanoparticles and a novel cell-free therapy for bone regeneration. We discuss the recent advance in the use of synthetic liposomes and extracellular vesicles as bioactive materials combined with scaffolds, highlighting major challenges and opportunities for their applications in bone regeneration. We put a particular focus on strategies to integrate vesicles to various biomaterial scaffolds and introduce the latest advances in achieving sustained release of bioactive molecules from the vesicle-loaded scaffolds at the bone defect site.

## 1. Introduction

With a rapidly aging global population, an increasing number of patients are suffering from bone diseases that need therapeutic intervention at defect sites [1]. The primary treatment for bone defect reconstruction has been bone grafting, mainly autografts or allografts. However, there exist clinical complications in current bone grafts, such as limited availability and donor site morbidity for autografts, or unwanted disease transmission and immunological reaction for allografts [2,3]. Bone tissue engineering (BTE) has become a promising approach for bone graft substitutes, promoting bone regeneration without incurring risks involved in the aforementioned bone grafts [4]. The BTE system comprises three major components to replicate the microenvironment for bone regeneration: biomaterial scaffold, cells, and biologically active (bioactive) molecules. The ideal BTE system aims to orchestrate all components to enhance bone repair and regeneration [4,5]. As several bioactive molecules including growth factors, nucleic acids, peptides, and small molecules have been demonstrated to play important roles in regulating cellular activities and bone tissue development, numerous studies have applied bioactive molecules into the BTE scaffolds [6]. Nonetheless, successful delivery of bioactive molecules at the bone defect site has been hampered due to a lack of proper delivery systems, giving rise to fast degradation, burst release, and non-specific targeting of bioactive molecules [7,8]. Accordingly, the regenerative performance of current BTE scaffolds requires improvement for clinical practice, urging need for the development of efficient bioactive molecule delivery systems.

Herein, we review recent approaches for developing successful bioactive molecule delivery systems in local bone regenerative therapy, particularly through employing liposomal vesicles or extracellular vesicles as drug delivery systems (DDS). In recent years, liposomes or extracellular vesicles have been integrated into various BTE scaffolds via different strategies, in which bound vesicles aimed to exert controlled and sustained release of bioactive cargo accelerating bone regeneration at the local defect site [9,10,11,12]. Liposomes, nanovesicles composed of lipid bilayer(s), have been utilized as versatile DDS since their first discovery in the 1960s, largely because they are biocompatible and capable of incorporating both hydrophilic and hydrophobic molecules [13]. One of the advantages of liposome carriers is that the surface of liposomes can be easily modified to carry specific targeting ligands for cell recruitment and/or to anchor to the biomaterial scaffold maintaining the long-term bioactivity of cargo [9]. The liposome-integrated scaffolds in bone regenerative applications will be reviewed in Section 2 with a focus on the functionalization of liposomes, types of cargo, and integration strategies to scaffolds.

Extracellular vesicles (EVs) are natural lipid-bound vesicles released by almost all types of cells, carrying many constituents of a parent cell, including DNA, RNA, metabolites, etc. [14]. Due to their inherent capacity to carry bioactive molecules, EVs are emerging as an advanced substitute for synthetic liposomes [15] as well as a novel cell-free therapy [16] for bone regeneration. Although ex-vivo cultivated cells have been considered an important component of BTE, a cell-free BTE system has recently begun to gain attention because it can avoid undesirable side effects such as limited autologous cells, low survival rate, tumorigenesis, and immune-rejection issues [16,17]. EVs as natural cargo carriers of their cellular origin or as engineered carriers of external cargo hold great potential in empowering endogenous regenerative properties of BTE systems, which will be discussed in Section 3.

The purpose of this review is to summarize the development of vesicle-integrated BTE scaffolds where vesicles convey bioactive molecules for efficient bone regeneration. We highlight synthetic liposomes and extracellular vesicles as an emerging component of BTE, discussing major challenges and opportunities for their applications in bone regeneration. We put a particular focus on strategies to integrate vesicles to various biomaterial scaffolds and introduce the latest advances on achieving sustained release of bioactive molecules from the vesicle-loaded scaffolds at the bone defect site.

## 2. Liposomal Vesicle-Integrated Scaffolds for Bone Regeneration

Liposomes are lipid-based nano-vesicles, assembled by emulsifying amphiphilic lipids such as phospholipids and cholesterols in the aqueous phase. Due to their amphiphilic properties, liposomes form an aqueous inner phase surrounded by lipophilic bilayer(s) that can encapsulate hydrophilic molecules in the inner phase and hydrophobic molecules in the bilayer(s). Liposomes have been widely applied as DDS to deliver chemicals, proteins, peptides, and genetic molecules because of their biocompatible nature and capability to accommodate high content of payloads protecting from degradation [13,15]. There exist commercial liposomal therapeutics in clinical applications, indicating excellent biocompatibility and versatility as DDS [13,15].

Biomaterial scaffolds incorporating liposomes have been developed to synergize therapeutic effects and mechanical supports for efficient bone regeneration. Loading liposomes, proven as widely applicable DDS, into BTE scaffolds can bring benefits such as the solubilization and stabilization of bioactive cargo, increasing the bioavailability and retention of cargo for improved efficacy [9]. Existing systems, however, need further improvement for control over the sustained release of cargo into the bone microenvironment for enhanced boner repair [9,13].

We discuss recent efforts to functionalize liposomes for controlled cargo release in Section 2.1 and various types of bioactive cargo loaded into liposomes and BTE scaffold systems for bone repair in Section 2.2. Conventional liposomes do not adhere to BTE scaffolds, urging surface modification engineering approaches. We summarized the surface modification approaches used in liposome-integrated scaffolds in Section 2.3. In Section 2.4, we explained the signaling pathways explored in applications of liposome-integrated scaffolds for local bone regenerative therapy.

### 2.1. Functionalization of Liposomes

**Figure 1:**
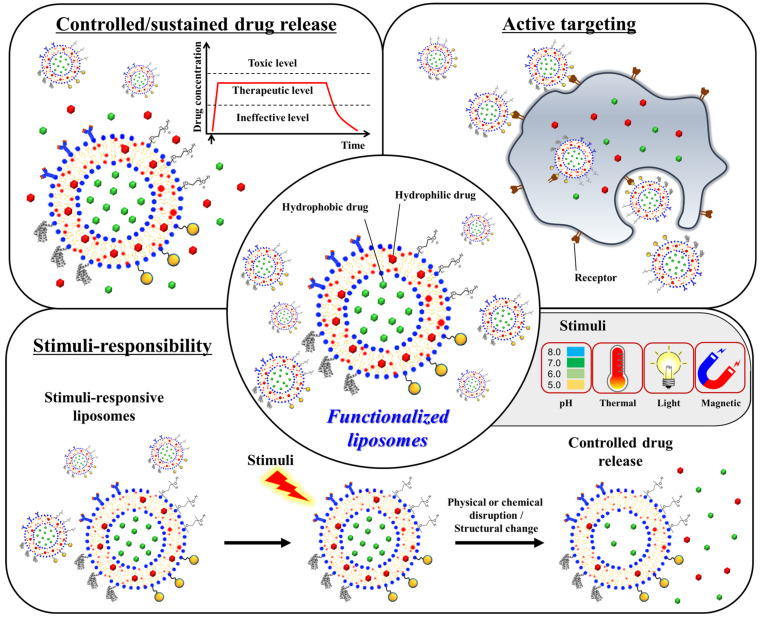
Scheme of functionalized liposomes used in bone regeneration applications. For efficacious bone repair, liposomes have been tailored to release drugs in a controlled manner, to hold active targeting moieties, and to be stimuli-responsive.

Herein, we will introduce the functionalization strategies applied to conventional liposomes for efficacious bone repair (Figure 1). For enhanced bone regeneration, liposomes have been tailored to release drugs in a controlled manner, to hold active targeting moieties, and to be stimuli-responsive.

#### 2.1.1. Thermosensitive Liposomes

Conventional liposomes lack the capability of controlling on-demand cargo release, limiting their therapeutic utility [13]. In order to provide on-demand delivery of therapeutics, thermosensitive liposomes (TSLs) have been developed to exert spatial and temporal control of therapeutic cargo upon thermal stimulus for localized bone regenerative therapy [18,19]. TSLs contain thermo-responsive phospholipids, such as dipalmitoylphosphatidylcholine (DPPC) and distearoylphosphatidylcholine (DSPC), which are saturated fatty acids with the transition temperature of 41.3 and 55.6 °C, respectively. The high transition temperature of thermo-responsive phospholipids enables TSLs to transform from a solid gel phase to a permeable liquid phase at a slightly higher temperature than the body temperature, allowing the controlled release of encapsulated therapeutics upon the external thermal stimulus.

López-Noriega et al. developed TSLs comprising DPPC, 1-monostearoyl phosphatidylcholine (MSPC), 1,2-distearoyl-sn-glycero-3-phosphoethanolamine-n-[methoxy (polyethylene glycol)-2000] (DSPE-PEG2000), and DSPE-PEG2000-maleimide with molar ratio 86:10:2:2 to deliver PTHrP 107–111, a pentapeptide with pro-osteogenic and anti-osteoclastic activity for bone regeneration [18]. These TSLs were bound onto the surface of a biocompatible collagen-based scaffold by covalent interaction between thiol groups on the scaffold and maleimide groups on the TSLs, giving rise to the thermo-responsive drug-eluting scaffold. The presence of TSLs showed no negative effects on the attachment and proliferation of pre-osteoblastic cells. Under the external thermal stimulus, the release of PTHrP 107–111 was shown to be accelerated from TSLs. The pro-osteogenic effects arising from the controlled delivery of PTHrP 107–111 were demonstrated by examining the alkaline phosphatase activity and expression of osteogenic genes in an osteoblast precursor cell line (MC3T3-E1).

Liu and coworkers developed a thermo-responsive hydrogel with liposome for local therapy of bone tuberculosis [19]. This injectable hybrid liposome-in-hydrogel system was developed from phospholipid-based liposomes and thermo-responsive PLGA-PEG-PLGA copolymers for sustained delivery of N′-dodecanoylisonicotinohydrazide (DINH), a drug for tuberculosis therapy. The hybrid hydrogel was shown to be thermo-responsive, self-healing, and capable of sustained drug release, demonstrating its potential use for the efficient long-term treatment of bone tuberculosis.

#### 2.1.2. Adhesive Liposomes

The retention of bioactive molecules in the target lesion dictates drug efficacy in vivo. To achieve prolonged retention of bioactive molecules at the bone defect sites, liposomes that are adhesive to the bone microenvironment or BTE scaffolds have been developed [20,21,22].

Liu et al. reported adhesive liposomes using cationic lipid, octadecylamine, for delivery of BMP-2 [20]. The PEG hydrogels containing adhesive liposomes were administrated locally to the bone defect sites. The liposomes released from hydrogels were shown to adhere to the bone defects via non-specific electrostatic interactions between positively charged liposomes and negatively charged moieties of local tissue and cells, yielding enhanced bone regeneration. In vitro biocompatibility, adhesiveness, osteogenic activities, and in vivo bone healing in osteoporosis and fracture models were evaluated and reported with good osteogenic activity and efficacious bone treatment.

Bisphosphonate and its derivatives, such as alendronate, can bind to calcium ions in the bone and accumulate to a high concentration in the bone. By taking advantage of the interactions between bisphosphonate and bone mineral, Wang et al. developed bisphosphonate-functionalized liposomes (BP-liposomes) and hydroxyapatite-coated collagen (Col/HA) scaffolds decorated with BP-liposomes on the surface [21]. These BP-lipo-Col/HA scaffolds exhibited a sustained release of various types of drugs including carboxyfluorescein, doxorubicin, and lysozyme, suggesting the potential to be used as controlled drug delivery systems for enhanced bone regeneration. Recently, Zhang et al. reported the alendronate-functionalized liposomes incorporating oxidized cholesterol derivatives, named oxysterol, and sonic hedgehog (Shh) encoding gene for bone healing via activation of hedgehog signaling [22]. These liposomes can bind onto the hydroxyapatite-coated PLGA scaffold and have a potential for extended retention in bone tissue through the interaction between alendronates and calcium ions in bone ECM. Furthermore, oxysterols can bind to a smoothened receptor on the membrane of bone cells, resulting in up-regulation of osteogenesis and ossification via the hedgehog signaling pathway. The scaffolds promoted the osteogenic differentiation of mesenchymal cells for bone regeneration in vitro and bone healing in the calvarial bone defect model in vivo.

#### 2.1.3. Bone-Targeting Liposomes

Active targeting can be achieved by exploiting specific binding between receptors and ligands where ligands are often antibodies, nucleic acid sequences, or peptides. By utilizing certain ligands, liposomes can be functionalized to be *bone-targeting* yielding efficient and precise bone repair. Most bone-targeting liposomes were designed based on the binding interactions between cationic moieties and negatively charged phosphates in bone tissues.

Bone-targeting liposomes with a targeting moiety, phosphorylated cholesterol, were developed for accelerated healing of the bone fracture [23]. The local delivery of salvianic acid A, a bone anabolic agent, along with the bone-targeting liposomes targeting bone fracture site, significantly enhanced the interaction with the bone, the retention of the liposomes in the bone, and the fracture healing in vivo. In recent study, Zhou et al. reported the collagen sponge incorporated with bone-targeting liposomes formed by phosphorylated cholesterols loaded salvianic acid A for endochondral ossification [24]. The critical-sized segmental defect rabbit model was assessed to determine the effect of bone regeneration via regulation of histone deacetylase 3 (HDAC3), which is related to the formation and development of the bone extracellular matrix (ECM). Micro-computed tomography (CT) analysis, histology, and immunofluorescence analysis strongly supported great potential of the bone-targeting liposome-incorporated scaffolds as an effective strategy for nonunion bone defect treatment.

Xu et al. developed the xenograft calcine bone scaffold combined with bone-targeting cationic liposomes [25]. The bone targeting cationic liposomes containing dioleoyl trimethylammonium propane (DOTAP) were further modified with repeated sequences, aspartate, serine, serine ((AspSerSer)6) (DSS)6 for delivery of CKIP-1 siRNAs. The introduction of (DSS)6 as a targeting ligand on the liposome enabled the localization of liposomes at the desired implanted region. The scaffolds promoted osteoblast proliferation and osteogenic differentiation with no influences on cell toxicity and activity. However, the ectopic ossification did not induce repairing of bone defect with the bone-targeting liposome-combined calcine bone scaffold, strongly supporting the results of pathological evaluations of defect, immunohistochemistry analysis, and micro-CT observation. In addition, the bone-targeting gene delivery system consisting of lipid, nucleic acid, and bone-homing peptide (DSSDSSDSSCSSDSSDSSD) was reported by Vhora and coworkers for osteoporosis treatment post-intravenous administration [26].

#### 2.1.4. Osteoinductive Liposomes

Conventional liposomes generally contain high contents of non-bioactive lipid components such as phospholipids and cholesterols which lack intrinsic bone regenerative properties. To improve conventional liposomal formulations, our group has developed an osteoinductive liposomal formulation by utilizing oxysterols as a component of liposomes [27]. Oxysterols are a class of steroid derivatives known to stimulate osteogenesis and ossification [28]. We incorporated 20S-hydroxycholesterol, one of the most potent oxysterols for bone regeneration, into non-phospholipid liposomes composed of stearylamine (SA), a single-chain amphiphile. The incorporation of 20S-hydroxycholesterol into SA-liposomes successfully induced in vitro osteogenesis and in vivo calvarial defect healing when localized to the methacrylated glycol chitosan (MeGC) hydrogel scaffold [27]. Recently, the potential of those osteoinductive liposomes as a drug carrier was further examined by delivering osteogenic molecules such as purmorphamine, smoothened agonist (SAG), and signaling molecule Shh [22,29,30]. The combined delivery of several osteogenic molecules achieved the enhancement of osteogenesis and in vivo bone repair.

### 2.2. Cargo Loading into Liposomes

In this section, we review various types of cargo incorporated into liposomes for bone regeneration applications. Proteins, peptides, hydrophilic/hydrophilic chemical agents, and genes have been applied to the liposomes.

#### 2.2.1. Proteins and Peptides

Bone morphogenetic protein-2 (BMP2) has been extensively used to treat nonunion bone defects in clinical applications. However, repetitive use in large doses of BMP2 is required to achieve the desired bone healing effect and to mitigate diffusional clearance. Liposomes have also been used for on-demand BMP2 delivery. For example, recombinant human BMP2 (rhBMP2)-incorporated magnetic liposomes composed of egg phosphatidylcholine (PC), cholesterol, and iron oxide nanoparticle (mean diameter of 10 nm) were reported by Matsuo et al. for bone healing in a rat bone-defect model post-topical injection into the defects [31]. Crasto et al. utilized liposomes to deliver rhBMP2 in a controlled release manner upon ultrasound stimuli, which provides a post-treatment control strategy in broader bone tissue engineering applications [32].

Liposomes were developed for sustained delivery of BMP2 peptides up to 21 days. The release profile regulation of the BMP-2 peptide is achieved by covalent immobilization on the hydroxyapatite nanoparticle-coated nanofibrous poly L-lactic acid scaffold [33]. These scaffolds initiated locally ectopic bone formation. López-Noriega et al. developed a thermo-responsive liposomal formulation for delivering pro-osteogenic pentapeptide, PTHrP 107–111 [18]. The thermo-responsive liposomes were covalently attached to the collagen-hydroxyapatite scaffold by introducing thioether bonds between maleimide groups on the liposome and thiol groups on the scaffold. Those scaffolds showed on-demand release of the peptides upon thermal stimuli (42 °C), which induced pro-osteogenic effects [18].

#### 2.2.2. Hydrophobic Small Molecule Drugs

Various hydrophobic small molecular drugs, including curcumin, aspirin, oxysterols, purmorphamine, and smoothened agonist (SAG), have been extensively applied to the liposomal delivery systems for bone tissue engineering. The focus has been improving the bioavailability of hydrophobic small molecular drugs against their poor solubility in an aqueous phase, rapid metabolism, and rapid systemic clearance. Sarkar et al. reported curcumin-loaded liposomes combined with a three-dimensional calcium phosphate scaffold that supports human fetal osteoblasts (hBOF) and positive osteogenic activity and also showed cytotoxic effect against human osteosarcoma cells (MG-63) [34].

Aspirin is known as a nonsteroidal anti-inflammatory drug (NSAID) with protective effects of the bone microenvironment in the survivor and differentiation of osteoblasts and their precursor cells. However, concerns for resistance and toxicity by high-dose and long-term use in clinical applications require advances with delivery systems, particularly, liposomes to achieve reduced toxicity, controlled release, and extended therapeutic period. Li and coworkers introduced an aspirin-laden liposomal delivery system, and its composite PCL scaffold enhances in vitro osteo-promoting proceedings of human mesenchymal stem cells and consequently induces in vivo ossification post-subcutaneous administration in a rat model [35]. This group also proposed a combined PCL scaffold with aspirin-encapsulated liposome and bone-forming peptide-1 (BFP-1) accelerated osteogenic efficiency in vitro and bone defect repair in vivo via PI3K/AKT pathway [36].

Our group reported an osteoinductive liposomal delivery system, named sterosome, which is formed from a high contents of cholesterol, with oxysterol as an osteoinductive cholesterol [27]. The integrated hydrogel scaffold with osteoinductive sterosomes showed excellent osteogenic activity with biocompatibility in vitro and effective bone healing in vivo in non-healing calvarial defect model. The loading of osteogenic hydrophobic small molecules (purmorphamine, SAG) in the osteogenic sterosomes and further hybrid designs of sterosome-coated PLGA scaffolds showed combinatory and synergistic pro-osteogenic events in vitro and in vivo re-ossification [29,30].

The application of kartogenin limits in the clinic, especially, intraarticular delivery because of its poor solubility. Gelatin-based composite hydrogel with liposome was reported to deliver kartogenin, which is a hydrophobic heterocyclic compound and stimulates chondrogenesis for osteoarthritis treatment [37]. The gelatin hydrogel microgels extended the release of kartogenin for over three weeks in vitro and the residence time for over five weeks in joint in vivo. Totally, the in vitro and in vivo evaluations showed that the microgel promoted cartilage regeneration and prevented osteoarthritis progression.

#### 2.2.3. Hydrophilic Small Molecule Drugs

Several liposomal systems for hydrophilic small molecule drug delivery have been investigated regarding controlled/sustained delivery against rapid diffusional clearance. Liposomal formulations containing salvianic acid A, as a potent inducer of osteogenesis and angiogenesis, combined with collagen scaffolds successfully stimulated bone fracture healing in mice and rabbit model [23,24].

Furthermore, various types of scaffolds, such as hydrogel and 3D printed scaffold, were developed for delivery of deferoxamine (DFO), an FDA-approved hydrophilic drug for the treatment of iron poisoning. In bone tissue engineering, the potential of DFO is known to promote angiogenesis and bone remodeling. The delivery of DFO by liposomal formulation showed controlled release of DFO in the composite hydrogel [38]. The composite of liposomes in the photo cross-linkable hydrogel of gelatin methacrylate also enhanced mechanical properties including rheology, compression, and stretching tests. The results based on the osteogenesis and angiogenesis in both in vitro and in vivo experiments strongly indicated efficacious promotion of the osteogenesis and angiogenesis differentiation both in vitro and in vivo. Very recently, the composite scaffold with 3D printed β-tricalcium phosphate (β-TCP) scaffold and hydrogel microsphere, which incorporated DFO-loaded liposomes, was reported by Han and coworkers [39]. This composite scaffold of 3D printed bioceramic scaffold and liposome-laden microgel promoted the internal vascularization and the osteogenic differentiation of stem cells. Conclusively, the composite scaffold promotes the mineralization and mature bone ingrowth for bone regeneration.

#### 2.2.4. Genes

Liposomes as a non-viral vector are employed for the delivery of nucleic acids. In particular, the limitations of viral vectors hastened the emergence of non-viral gene transfer with liposomal formulations. Liposomal gene delivery enables rational design of gene delivery to overcome critical biological challenges, including physicochemical stability, cellular internalization, endosome escape, and nuclear localization [9].

Generally, a number of liposomes containing cationic lipids and/or other cationic compounds were developed as condensation agents of genes (DNAs or RNAs) via electrostatic interactions to load. Monteiro et al. reported a gene delivery device, combining liposomes for delivery of runt-related transcription factor 2 (RUNX2) genes and polycarprolactone nanofibrous scaffolds [40]. The scaffold was functionalized with RUNX2 gene-loaded liposomes on their surface by a thioether linkage between maleimide and thiol groups. This approach achieved a long-term gene expression and induction of osteogenic markers in human bone-marrow-derived mesenchymal stem cells, promoting osteogenic differentiation for bone tissue engineering. Cationic non-phospholipids liposomes were developed to deliver small interfering RNA (siRNA) targeting noggin, which is an antagonist of BMP [41]. These liposomes showed a colloidal stability and promoted efficacy of cellular internalization in comparison with commonly used lipofectamine (Invitrogen). The evaluations with in vitro 2D monolayered cell and hydrogel-based 3D cell culture showed suppression of noggin expression by noggin siRNA-loaded liposomes, resulting in the enhanced osteogenic differentiation of adipose-derived mesenchymal stem cells. Furthermore, in vivo results supported this non-viral gene delivery system that promoted nonunion bone defect regeneration. Cui et al. also reported a simultaneous delivery of small molecular stimulator and noggin siRNA for regulation of BMP signaling with liposomes to enhance osteogenic differentiation of mesenchymal stem cells and re-ossification of mouse calvarial defect model [42]. Zhang and colleagues proposed a liposomal formulation including agonists of the hedgehog signaling pathway for efficacious bone healing [22]. The nanoparticulate agonists are consisted of oxysterols and complexes of polyethyleneamine/plasmid DNA encoding Shh, resulting in synergistically enhanced hedgehog signaling activation. This formulation showed a highly efficient expression of the Shh gene increased cellular internalization, endosomal escape, and nuclear localization with no significant cytotoxicity. Furthermore, the surface decoration of alendronate on the liposomes eased the integration of liposomes onto the apatite-coated 3D PLGA scaffold. The careful design of gene-carrying liposomes integrated with biomaterials successfully induced in vitro and in vivo efficacious osteoinduction.

### 2.3. Integration of Liposomes into Scaffolds

Various biomaterial scaffolds have played a crucial role in bone tissue engineering. The biomaterial scaffolds incorporating liposomes have been extensively explored for injured and diseased bone treatment (Table 1). Liposomes have received great attention as a biocompatible reservoir system for the protection and solubilization of drugs due to ease of functionalization and controlled delivery [9]. Rational design integrating advantages of liposomal delivery systems and 2D/3D architecture of biomaterial scaffolds provide an excellent microenvironment to regulate bone regeneration processes. Various drug-encapsulated liposomes can be incorporated into biomaterial scaffolds via physical, covalent, and non-covalent interactions for further applications in bone tissue engineering. The liposomes were generally incorporated by non-specific encapsulation in the pores of hydrogels [27,29,30,37,41,42]. Various dried state biomaterial scaffolds commonly incorporated the liposomes via electrostatic interactions [21,22,25,34,43]. However, non-specific interactions between liposomes and scaffolds often led to fast release or degradation of liposomes, failing to provide a sustained release of drugs incorporated inside the liposomes. To overcome such shortcomings, covalent cross-linking methods, such as a thioether linkage, were introduced [18,33,40,44]. Particularly, polydopamine-mediated layer-by-layer coating via Schiff base formation and Michael-type addition has been proposed to establish the stable binding of liposomes to the biomaterial scaffold [29,30,35,36]. The polydopamine-mediated coating approaches are highly biocompatible, chemical catalyst-free, and byproduct-free in comparison to conventional covalent cross-linking methods [45], demonstrating their potentials in building advanced liposome-integrated scaffolds for local bone regenerative therapy.

### 2.4. Signaling Pathways Explored in Liposome-Integrated Scaffolds

**Figure 2:**
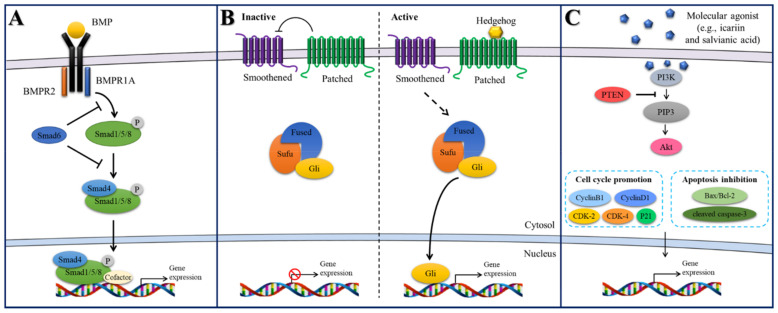
Schematics of signaling pathways explored in liposome-integrated scaffolds. (**A**) BMP/Smad signaling pathway. (**B**) Hedgehog signaling pathway. (**C**) PI3K/Akt signaling pathway.

As reviewed in previous sections, liposomes in BTE have been employed to carry and deliver various bioactive molecules to the bone microenvironment. Such bioactive molecules exert their bone regenerative efficacy via different signaling pathways, such as BMP, Hedgehog, Wnt, or RNAKL-RANK signaling [46]. In the following section, we review and discuss signaling pathways explored in liposome-integrated scaffolds for bone regeneration applications (Figure 2).

#### 2.4.1. BMP/Smad Signaling

BMPs regulate differentiation of mesenchymal progenitors into chondrocytes and osteoblasts. BMPs are critical for embryogenesis, cell growth, and adult homeostasis [47]. The BMP signaling pathway is initiated by serine–threonine kinase transmembrane receptors through glycoprotein effectors called Smads regulating intracellular processes and transcriptional mechanisms [48]. BMP/Smad signaling also links pathophysiological events related to bone disease and remodeling. The effects for bone remodeling and repair of various molecular activators, including BMPs, BMP peptides, and phenamil, have been revealed, when designing with liposomal vehicles, even combined with certain biomaterial hydrogel and synthetic polymer scaffolds [20,33,42]. Furthermore, knockout of BMP antagonists using liposomal vector encapsulating siRNAs showed promoted osteogenic and bone healing effects via controlling BMP signaling pathway [41,42].

#### 2.4.2. Hedgehog Signaling

Hedgehog signaling plays a crucial role in bone tissue/organ development. Shh, Indian hedgehog, and desert hedgehog belong in the hedgehog family in vertebrates [22]. The hedgehog signaling is regulated by transmembrane proteins, such as smoothened and patched. Patched suppresses the activity of smoothened in the rest stage [30]. The signaling is activated by turning the inhibition activity of patched hedgehog for suppression of smoothened. The activation of smoothened initiates downstream events, leading to the osteogenesis. Thus, osteogenesis is promoted by treatment and delivery of hedgehog encoding gene and hedgehog agonists, such as purmorphamine, smoothened agonist, and oxysterols, regulating positively hedgehog signaling pathway) [22,27,29,30].

#### 2.4.3. PI3K/Akt Signaling

Phosphatidylinositol 3-kinase (PI3K) is a member of the lipid kinase family that is an upstream of the serine/threonine kinase Akt. Signaling pathway of PI3K/Akt is related to the regulation of physiological events, including adhesion, proliferation, differentiation, and metabolism of cells [49]. The PI3K/Akt signaling plays a pivotal role in the regulation of osteoblasts and osteoclasts fates and thus bone tissue metabolism including preventing and reducing osteoporosis [50,51]. Small molecular compounds (icariin and salvianic acid) are also explored to treat bone-related disease, such as osteoporosis [52]. In particular, the liposome-integrated scaffold combined with dual-bioactive agents, aspirin and bone forming peptide-1, activated the PI3K/Akt signaling pathway, resulting in efficacious re-ossification [36].

## 3. Extracellular Vesicle-Integrated Scaffolds for Bone Regeneration

Almost all types of cells secrete EVs into the extracellular space, as part of their modes of intercellular communication [53]. EVs are the collective term for different types of vesicles including exosomes and ectosomes. Exosomes are a subset of EVs with an endosomal origin and a size range of 40–160 nm [53]. The International Society for Extracellular Vesicles (ISEV) urged the use the operational terms for EV subtypes such as “small EVs” since consensus on specific markers of EV subtypes has not yet been established [54]. However, we noted that most reports used the term “exosomes” within their experimental systems. In this review, we used the term “exosomes” when the authors specified the definition of exosomes in their reports. When not clearly noted, we used the general term “EVs”. 

EVs by themselves or as engineered carriers for the delivery of payloads are an active area of research in the field of bone regenerative medicine [10,11,12]. Compared with liposomes, EVs offer more excellent biocompatibility, efficient cellular internalization, and specific tissue targeting capability [14,15]. Many constituents of exosomes reflect their cellular origin, including nucleic acids, proteins, lipids, and metabolites [53,55,56]. Notably, exosomal miRNAs delivered into recipient cells can effectively initiate the signal cascade, altering biological activities of the recipient cells [57]. In Section 3.1, we review studies where EVs derived from various cell lines exert a positive effect on bone regeneration by delivering bioactive molecules originated from their parent cells. In Section 3.2 and Section 3.3, we introduce the approaches to engineer exosomes to harness their therapeutic potential in local bone regenerative therapy. In Section 3.4, we elaborate on strategies to integrate EVs into biomaterial scaffolds.

### 3.1. Source and Function of Extracellular Vesicles 

Numerous studies have focused on revealing the functional role of exosomes on bone regeneration both in their naïve and engineered form [10,11,12]. In Section 3.1, we summarize the current understanding of osteoinductive potentials of exosomes isolated from different parental cell sources. Exosomes modified by either endogenous or exogenous engineering will be elaborated in Section 3.2 and Section 3.3, respectively.

#### 3.1.1. Bone Cells

Table 2 summarizes the previous studies in which the effects of bone cell-derived EVs were explored in local bone regenerative therapy. Bone cells in Table 2 include bone marrow mesenchymal stem cells (BMSCs), pre-osteoblasts, and osteoclasts.

MicroRNA (miRNA) profiling for the BMSC-derived exosomes have shown that nine miRNAs (let-7a, miR-199b, miR-218, miR-148a, miR-135b, miR-203, miR-219, miR-299-5p, and miR-302b) are up-regulated, and four miRNAs (miR-221, miR-155, miR-885-5p, miR-181a, and miR-320c) are down-regulated during the osteoblastic differentiation of human-derived BMSCs (hBMSCs) [58]. These data demonstrate that exosomal miRNAs in BMSC-derived exosomes are important post-transcript regulators of osteogenesis and bone remodeling, informing the therapeutic potential of exosomes in bone repair. Numerous studies have revealed that BMSC-derived exosomes exert an osteoinductive effect in animal models of bone defects via different proposed pathways (refer to Table 2 for details).

The macrophage-osteoclast axis is an essential component of bone remodeling and resorption [59]. A few studies have investigated the osteo-immunomodulatory role of exosomes derived from osteoclast lineage in bone regeneration [60,61,62]. Liang et al. [62] showed that exosomes derived from mature osteoclasts promote osteogenesis to recipient rat-derived BMSCs (rBMSCs) in vitro and further verified the pro-osteogenic activities of the exosome-coated demineralize bone matrix (DBM) in mouse calvarial defects in vivo. Rat bone marrow macrophages were stimulated with macrophage colony-stimulating factor (M-CSF) and receptor activator of nuclear factor kappa-Β ligand (RANKL). Such mature macrophage-derived exosomes were shown to secrete miR-324 that silences ARHGAP1, a negative regulator of osteogenic differentiation, thereby facilitating bone defect healing through ARHGAP1/RhoA/ROCK signaling [62].

#### 3.1.2. Other Stem Cells

Besides osteoblast or osteoclast lineage, various types of stem cells have been explored in exosome-based bone regeneration applications. Exosomes derived from adipose-derived stem cells (ASCs), umbilical cord mesenchymal stem cells (UCMSCs), induced pluripotent stem cells (iPSC), etc. demonstrated osteogenic potential in preclinical animal models as summarized in Table 3. The hASC-derived exosomes were shown to promote the proliferation, migration, and osteogenic differentiation of hBMSCs in vitro [63,64] and enhance bone regeneration in vivo [63,64,65,66]. Chen et al. [65] conducted gene network analysis and showed that hASC-EVs contain multiple osteoinductive miRNAs such as miR-21, let-7f, miR-10a, miR-10b, and miR-199b, which may explain the osteogenic capacity of hASC-EVs.

Zhang et al. [67] reported that exosomes derived from hUCMSCs enhanced bone repair through hypoxia-inducible factor 1- α (HIF-1α)-mediated promotion of angiogenesis in a rat model of femoral fracture. HIF-1α was shown to regulate the hUCMSC-exosome-mediated expression of vascular endothelial growth factor (VEGF). The pro-angiogenic effect of hUCMSC-exosomes was exerted to the localized fracture sites via administration of commercial hydrogel carrying exosomes. The same group revealed in a different study that miR-21 in hUCMSC-exosomes is a potential intercellular messenger that promoted angiogenesis via upregulating the NOTCH1/DLL4 pathway.

**Table 2:** Bone cell-derived EV-integrated biomaterial scaffolds for bone regeneration.

Source of Exosomes	Pre-conditioning	Scaffold	In Vivo Model	Function of Exosome-Integrated Scaffolds	Reference
rBMSCs	OM	Alginate-PCL	Nude mouse subcutaneous bone formation model	Pro-angiogenic and pro-bone regeneration activities in vitro and in vivo.	[68]
rBMSCs	OM	Decalcified bone matrix (DBM)	Nude mouse subcutaneous bone formation model	Pro-angiogenic and pro-bone regeneration activities in vitro and in vivo.	[69]
hBMSCs	-	Gelatin methacrylate (GelMA)	Rabbit osteochondral defect model	Enhanced mitochondrial biogenesis in vitro and facilitated cartilage regeneration in vivo.	[70]
hBMSCs	Hypoxia	Commercial HA scaffold	Rat calvarial defect model	Pro-angiogenic activities via AKT/mTOR pathway.	[71]
hBMSCs	OM, Noggin Suppression	Injectable chitosan hydrogel	Mouse calvarial defect model	Elevated osteogenesis via inhibition of miR-29a in BMP/Smad signaling.	[72]
hBMSCs	-	DBM	Rat subcutaneous implantation and Rabbit femoral condyle bone defect model	Human BMP2 plasmids were coated onto the vesicles. Promoted bone formation and angiogenesis in animal model.	[73]
hBMSCs	-	Calcium sulfate/ nanohydroxyapatite based nanocement	Femur neck canal defect model in osteoporotic rats	Enhanced bone formation in the absence of BMP.	[74]
hBMSCs	OM	Titanium alloy	Rat radial bone defect model	Promoted osteogenic differentiation via PI3K/Akt and MAPK signaling pathways.	[75]
rBMSCs	OM	Mesoporous bioactive glass (MBG)	Rat calvarial defect model	Osteoinductivity attributed to exosomal miRNAs (let-7a-5p, let-7c-5p, miR-328a-5p, and miR-31a-5p).	[66]
hBMSCs	-	Titanium alloy	Osteoporotic bone defect model	miR-20a in hBMSC-EVs was shown to play a key role in promoting osteogenesis.	[76]
hBMSCs	BMP2 overexpression	Alginate-RGD hydrogel	Rat calvarial defect model	EVs were tethered to biomaterial scaffolds with ECM proteins, which promoted bone repair and prolonged delivery in vivo.	[77]
Preosteoblasts MC3T3	-	Alginate hydrogel	-	Verified the osteogenic potential of MC3T3-derived EVs in vitro.	[78]
Murine-derived macrophage	BMP2 stimulation	Titanium dioxide nanotubes	-	BMP2/macrophage-derived exosomes enhanced the osteogenic differentiation of MSCs in vitro.	[60]
Osteoclast from osteoporotic rats	-	Magnetic nanoparticle-infiltrated hydroxyapatite	-	The presence of magnetic nanoparticles altered osteoclast-derived exosomal cargo and decreased the uptake efficiency of osteoclast exosomes in osteoblasts.	[61]
Osteoclast from mice	-	DBM	Rat calvarial defect model	Elevated osteogenesis via miR-324 in ARHGAP1/RhoA/ROCK signaling.	[62]

rBMSCs: rat-derived bone marrow stem cells; hBMSCs: human-derived bone marrow stem cells; OM: osteoinductive medium; BMP: bone morphogenic protein; RGD: Arginylglycylaspartic acid.

**Table 3:** Various stem cell-derived EV-integrated biomaterial scaffolds for bone regeneration.

Source of Exosomes	Pre-Conditioning	Scaffold	In Vivo Model	Function of Exosome-Integrated Scaffolds	Reference
hASCs	OM	Polydopamine-coated PLGA	Rat calvarial defect model	Promoted proliferation, migration, and osteogenic differentiation of hBMSCs in vitro. Enhanced migration and homing of hBMSCs in vivo.	[63]
hASCs	-	Biotin-doped polypyrrole titanium	Nude mouse subcutaneous bone formation model	Osteoinductive ability of hASC-EVs was evaluated via analysis of its content miRNAs.	[65]
hASCs	-	CaSi-coated PLA	-	Enhanced osteogenic properties in vitro.	[79]
hASCs	-	Silk fibroin	Rat calvarial defect model	Improved osteogenic differentiation of hBMSCs in vitro. Promoted the production of collagenous tissues and bone-like tissue in vivo.	[64]
Chondrogenic progenitor cells	-	Core-shell nanofiber film of CS/PLA	Rat calvarial defect model	VEGF plasmid DNA was sustainably delivered, resulting in elevated vascularized osteogenesis in vivo.	[80]
Chondrogenic progenitor cells	-	3D printed PCL scaffolds	Rat radial bone defect model	Osteogenic differentiation of hBMSCs in vitro. Vascularized bone regeneration in vivo.	[81]
hiPSCs	-	Commercial β-TCP	Rat ovariectomized model	Enhanced angiogenesis and osteogenesis under osteoporotic conditions.	[82]
hiPSCs	-	Commercial β-TCP	Rat calvarial defect model	Osteogenic differentiation of hBMSCs via PI3K/Akt signaling.	[83]
hUCMSCs	-	Commercial hyaluronan-heparin hydrogel	Rat model of femoral fracture	HIF-1-mediated promotion of angiogenesis.	[67]
hUCMSCs		CHA/SF/GCS/DF-PEG hydrogel	Rat femoral condyle bone defect model	Sustained delivery of exosomes at the bone defect sites.	[84]
hUCMSCs	-	Hyaluronic acid-alginate hydrogel with HAP	Rat calvarial defect model	Controlled delivery of exosomes at the bone defect sites.	[85]
hUCMSCs	-	Hyaluronic acid hydrogel combined with customized HAP/poly-ε-caprolactone	Rat calvarial defect model	miR-21 is a potential intercellular messenger that promoted angiogenesis by upregulating the NOTCH1/DLL4 pathway.	[86]
hGMSCs	-	PLLA scaffold	Rat calvarial defect model	Improved vascular network and osteogenic regeneration.	[87]
hDPSCs	OM	PLLA scaffold	Nude mouse subcutaneous bone formation model	PLGA-PEG-PLGA triblock copolymer microsphere Controlled release of Exos from scaffolds was characterized.	[88]
rDPSCs	-	Commercial collagen, β-TCP, or HA scaffold	Rat calvarial defect model	DPSC-EVs-loaded scaffold showed a comparable bone regeneration effect to the DSPC-loaded scaffolds.	[89]
hSHED	-	β-TCP	Rat periodontal defect model	Periodontal bone regeneration through AMPK signaling.	[90]

ASC: adipose-derived stem cells; UCMSC: umbilical cord mesenchymal stem cells; iPSC: induced pluripotent stem cells; OM: osteoinductive medium; PLGA: poly(lactide-co-glycolide); PLLA: poly(L-lactic acid); β-TCP: β-tricalcium phosphate; CHA: coralline hydroxyapatite; SF: silk fibroin; CS: chitosan; GCS: glycol chitosan; DF-PEG: difunctionalized polyethylene glycol; GMSC: gingival mesenchymal stem cell; SHED: stem cells from human exfoliated deciduous teeth.

### 3.2. Endogenous Engineering of Extracellular Vesicles

In the previous Section 3.1, we summarized the different sources of parent cells that yielded exosomes of similar features to their parent cells, having angiogenic or osteogenic potentials. Several studies have shown that not only the type of parent cell but culture conditions called “pre-conditioning” can determine the content of exosomes [91]. In this section, we introduce the methods of engineering exosomes at the cellular level by pre-conditioning parent cells in culture. Approaches to produce exosomes with enhanced angiogenic or osteogenic potentials are summarized with specific examples in local bone regenerative therapy.

#### 3.2.1. Pre-Conditioning Parent Cells to Enhance Angiogenic Potential

Liang et al. [71] preconditioned hBMSCs with a low dose of dimethyloxaloylglycine (DMOG) and examined the proangiogenic effects of those hBMSC-derived exosomes. DMOG is a small molecule inhibitor of prolyl hydroxylase which upregulates HIF-1α. Based on the previous finding that HIF-1α overexpressed hBMSCs enhanced angiogenesis [92], Liang et al. [71] tested whether exosomes collected from hypoxia-conditioned hBMSCs could exert similar proangiogenic effects as parent cells. Exosomes derived from DMOG-treated hBMSCs showed enhanced angiogenesis in vitro, which may be attributed to the downregulation of PTEN expression. PTEN is a well-defined tumor suppressor gene in which the downstream target pinpoints the AKT/mTOR pathway. For in-vivo study, DMOG-MSC-exosomes were integrated into the classical porous hydroxyapatite scaffolds and locally applied to rat calvarial defects. At 8 weeks post-surgery, enhanced bone regeneration with neovascularization was observed. This work represents a good example of modulating parental cells to yield *proangiogenic* exosomes, for the treatment of skeletal injury and tissue resection.

#### 3.2.2. Pre-Conditioning Parent Cells to Enhance Osteogenic Potential

**BMP2 Stimulation.** Bone morphogenic protein 2 (BMP2) is the only FDA-approved and most widely used growth factor in bone regeneration [93]. BMP2 treatment was reported to exert a positive immunomodulatory effect on macrophages [94] and potentiate the BMP2 signaling cascade on hBMSCs [77,95], favoring the local environment for enhanced bone regeneration. Huang et al. [95] demonstrated the enhanced bone regenerative potential of BMP2-treated hBMSC EVs compared to naïve hBMSC EVs in a rat calvarial defect model in vivo. Wei and colleagues studied the osteogenic potential of exosomes derived from BMP2-treated murine macrophages (RAW 264.7) in bone regeneration [60]. The BMP2/macrophage-derived exosomes were incorporated to Ti implants followed by hBMSC seeding for in vitro study, which accelerated the osteogenic differentiation of hBMSCs compared with untreated control exosomes. The pro-osteogenic potential of the BMP2-treated macrophages was transferred to the daughter exosomes, demonstrating the great promise of parental cell exosome engineering for bone regeneration.

**Magnetic Nanoparticles.** In work by Zhu et al. [61], hydroxyapatite ceramics incorporating magnetic nanoparticles were used as a scaffold material to co-culture osteoblasts and osteoclasts. The presence of magnetic nanoparticles altered the osteoclast-derived exosomal cargo, giving rise to less uptake of osteoclast-derived exosomes into osteoblasts weakening exosome-mediated communications between osteoblasts and osteoclasts. As a result, enhanced osteoblast proliferation was observed in MHA scaffolds compared with control HA scaffolds. The MHA up-regulated Rho kinase but down-regulated ubiquitination, ATP, and reactive oxygen species in osteoclast-derived exosomes in vitro.

**Noggin Suppression.** The clinical exogenous use of BMP2 is often limited by unwanted side effects such as ectopic bone formation, bone resorption, and inappropriate adipogenesis [93]. One alternative approach is to induce endogenous BMP signaling by downregulating noggin, a natural BMP antagonist [96,97,98]. Our group genetically modified hMSCs through noggin knock-down and extruded those noggin-suppressed hMSCs to obtain exosome-mimetics (EMs) [72]. The noggin suppression in hMSCs led to the endogenous accumulation of osteogenic molecules in EMs. The preconditioned EMs with enhanced osteogenic potency was then loaded into an injectable methacrylated glycol chitosan (MeGC) hydrogel. The EM-integrated MeGC hydrogel exhibited robust bone regeneration in mouse calvarial defects, mediated through the increased noggin siRNA and reduced miR-29a. This work shows that transmitting efficient therapeutic modalities (enhanced osteogenic properties) to exosomes can be directed via genetic engineering of the parental cells.

**Osteogenic Induction.** Culturing MSCs in an osteoinductive medium is a well-established method to potentiate the osteogenic differentiation of MSCs into osteoblasts. Many studies have revealed that exosomes isolated from osteoinductive-conditioned MSCs show enhanced osteogenic potency accordingly as in parental cells [63,66,68,69,72]. Systemic study of optimizing osteogenic exosomes has been reported by Liu et al. [66] in which exosomes were collected from different stem cell sources (rBMSCs/rASCs) and culture conditions (osteoinductive/common). The exosomes derived from rBMSCs cultured in an osteoinductive medium (BMSC-OI-exo) demonstrated the most significant osteoinductivity in BMSCs among experimental groups (2-fold increase in alkaline phosphatase activity to blank control) while exosomes produced by rASCs under common culture medium showed very limited osteoinductivity in vitro. Detailed bioinformatic analysis and pathway verification revealed that enhanced osteogenesis is attributed to up-regulated miRNAs (let-7a-5p, let-7c-5p, miR-328a-5p, and miR-31a-5p) in BMSC-OI-exo. The loading of BMSC-OI-exo into mesoporous bioactive glass (MBG) scaffold led to enhanced bone regeneration in rat cranial defects in vivo.

### 3.3. Exogenous Cargo Loading into Extracellular Vesicles

In Section 3.2, endogenous engineering of exosomes was introduced; exosomes were modified at the cellular level by genetically manipulating parent cells. In this section, a few studies on exogenous modification of exosomes will be highlighted in the field of bone regenerative therapy. Even though external cargo loading into exosomes has been actively investigated for cancer therapy due to their preferred tumor homing and adjustable targeting efficiency [99,100,101], we noticed it has not been a major focus in the field of localized bone regeneration therapy, but rather, accumulating angiogenic/osteogenic factors within exosomes through an endogenous manner has been favored as introduced in Section 3.2. One possible explanation is that there is a lack of understanding in the consequences of external cargo loading into exosomes. Exosomes already contain proteins, genetic lipids, and genetic materials including mRNA and microRNA (miRNA) in their lumen. However, it remains unclear whether external cargo loading into exosomes disrupts their colloidal stability, integrity, or innate bioactivity, provoking future quality assessment [102]. Despite these potential concerns in exogenous cargo loading into exosomes, recent studies have demonstrated that external cargo-loaded exosomes integrated to the 3D scaffolds can serve as an effective vehicle to transfer payloads to a local microenvironment, significantly promoting bone repair at localized sites [73,80,81].

In work by Liang et al. [73], plasmids encoding human bone morphogenetic protein 2 (pBMP2) were added to rat MSC-derived extracellular vesicles to improve the osteoinductivity of decalcifined bone matrix (DBM). The surface of EVs was coated with polyethyleneimine (PEI), a cationic polymer widely used for packaging negatively charged DNA, after which pBMP2 was added via layer-by-layer (LBL) self-assembly. Such simple LBL method effectively formed EVs-PEI/pBMP2 complexes with a diameter ranging between 100–1000 nm. Compared with PEI/pBM2 control groups, EVs-PEI/pBMP2 complexes exhibited higher transfection efficiency of BMP2 gene and low cytotoxicity to MSCs in vitro. Furthermore, the DBM integrated with EVs-PEI/pDNA complexes showed enhanced bone regeneration in vivo, both in rat subcutaneous implantation and rabbit femoral condyle bone defect model.

Zha et al. [80] designed a gene-activated matrix for bone repair which can locally release VEGF plasmid from the EM-bound chitosan/poly (lactic acid) scaffold. For a detailed description of the exosome integration strategy to scaffold, refer to Section 3.4.3. Since VEGF is a key mediator of angiogenesis and an important component of bone repair, its delivery through an osteoinductive scaffold has been pursued in bone regeneration applications [103]. However, direct delivery of VEGF growth factors as proteins involved several issues such as fast degradation, burst release, and unstable bioactivity [104]. Zha and co-authors bypassed the aforementioned problems by delivering recombinant pVEGF protected within rBMSC-derived EMs [80]. The VEGF plasmids were loaded into EMs via the electroporation method in which an electrical pulse generates temporary pores in the membrane increasing membrane permeability. The gene-activated matrix successfully promoted vascularized osteogenesis in rat cranial defects. The same group also delivered pVEGF as exosomal cargo, demonstrating effective vascularized bone regeneration in segmental bone defects [81].

### 3.4. Integration of Extracellular Vesicles into Scaffolds

Successful bone regeneration through the exosome-loaded scaffolds demands the long-term preservation, bioactivity protection, and sustained release of exosomes at the bone defect sites. The way exosomes are integrated into the biomaterial scaffolds critically dictates such bone regenerative potency of the exosome-loaded scaffolds in the localized treatment area. In this Section 3.4, we outline the current strategies to load exosomes to various biomaterial scaffolds and highlight recent advances in prolonging the release of exosomes for localized bone regenerative therapy.

#### 3.4.1. Post-Solution Adsorption

**Figure 3:**
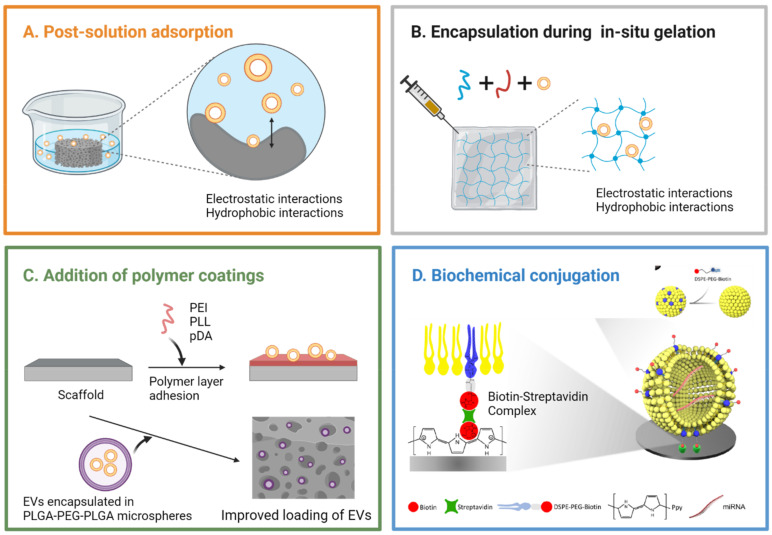
Schematics of different exosome loading methods to biomaterial scaffolds. (**A**) In case of post-solution adsorption, scaffolds are incubated in a solution containing exosomes, allowing physical adsorption of exosomes to the surface. (**B**) Exosomes can be loaded during in situ gelation of hydrogels, in which the pore network of hydrogel plays a critical role in retaining exosomes. (**C**) Addition of polymer layer onto biomaterial scaffold or encapsulation of exosomes inside polymer microspheres has been applied to advance the integration stability of exosomes to scaffolds. (**D**) Biotin-streptavidin complex was employed to better immobilize EVs onto the surface of titanium (Ti) scaffolds. The exosomal membrane as well as Ti scaffold surface were chemically modified to enable biotin-streptavidin binding. Panel (**D**) reproduced with permission of American Chemical Society [65]. Image produced with permission of BioRender.

Exosomes have been incorporated to various bioceramic, polymer, and composite scaffolds for bone regeneration applications including β-tricalcium phosphate (β-TCP) [82,83,90], DBM [62,69,73], polycaprolactone (PCL) [68,81], PLA [79,88], PLGA [63], mesoporous bioactive glass (MBG) [66], and titanium [60,75] among others. The most utilized method for incorporating exosomes to those scaffolds is to simply incubate scaffolds in a solution of exosomes allowing physical adsorption (post-solution adsorption) as illustrated in Figure 3A.

Qi et al. [82] and Zhang et al. [83] loaded exosomes to commercial β-TCP scaffolds through solution adsorption and demonstrated the pro-osteogenic effects of hiPSC-exosomes in a rat model of calvarial bone defects. However, the system showed an initial burst release behavior where 60% of the loaded exosomes were released on day 1, and complete release happened on day 5 in vitro, lacking the desired quality of controlled and extended release [83]. A similar exosome release profile was reported in SHED (stem cells from human exfoliated deciduous teeth)-derived exosomes/β-TCP studied for periodontal bone regeneration. More than 50% of the loaded exosomes were released from the β-TCP scaffold on day 1, and complete release happened on day 5, exhibiting a burst release [90].

Kim et al. [64] fabricated a highly porous silk fibroin (SF) scaffold using freeze-drying method and post-incubated hASC-derived exosomes to the scaffold. The release of exosomes was tracked for 7 days in vitro using micro-BCA assay and ELISA analysis on the expression of CD63. No initial burst release behavior was observed, and approximately 70% of the loaded exosomes were released for 7 days in a continuous manner. The exosome-releasing SF scaffolds promoted the recovery of critical-sized rat calvarial defects, accelerating formation of collagenous fibers and bone-like tissues at both 5 weeks and 10 weeks post-surgery. Liu et al. [66] constructed MBG scaffolds with micro-porosities (0.5–2 µm) and loaded rBMSC-derived exosomes to the scaffolds by dropping exosome concentrate. Such exosome-integrated MBG scaffold was lyophilized before use, and its bone repairing capacity was verified in a rat cranial defect model. Exosomes were rapidly released in the first week (60% cumulative release on day 7) followed by a steady release (approx. 75% release on day 28), which demonstrates a potential of preserving exosomes via lyophilization and its application in extending the release period of exosomes.

Not many studies reported the exosome release profiles from exosome integrated scaffolds, limiting further analysis. However, a few studies indicated the limitations of integrating exosomes to scaffolds by relying only on physical adsorption, which is not enough to retain exosomes for a desired bone regeneration period which usually takes several weeks. To have exosomes durably exert their biological efficacy at bone defect sites even after weeks of application, a more sustainable and controllable delivery method needs to be adapted.

#### 3.4.2. Encapsulation during In Situ Gelation

Various exosome-loaded hydrogels have been developed to preserve exosomes for an extended period, maintaining their local concentration at the treatment sites. Exosomes can be encapsulated inside the pores of hydrogels during cross-linking process (in situ gelation) as shown in Figure 3B. Recent literature reports that hydrogels in general exhibit more sustained release of exosomes than bioceramic scaffolds, which may be ascribed to the surface characteristics of engineered hydrogels including high porosity, excellent hydrophilicity, and adequate size of the pores for capturing exosomes. In this section, we will summarize recent applications of exosome-loaded hydrogels in bone regeneration. We will also highlight the surface modification strategies explored to better engineer the delivery of exosomes at bone defect sites in Section 3.4.3.

Qin et al. [105] utilized the commercial HyStem-HP hydrogel to encapsulate hBMSC-derived EVs during gelation. EVs were mixed with a combination of thiol-modified hyaluronan, thiol-modified heparin, and thiol-modified denatured collagen; then, the mix was crosslinked by the addition of a thiol-reactive crosslinker, poly(ethylene glycol) diacrylate (PEGDA). The release behavior of EVs was not tested in vitro, but hBMSC-EV-integrated hydrogels successfully stimulated bone formation in rat calvarial bone defects, which may be ascribed to the pro-osteogenic role of miR-196a in hBMSC-EVs. Yang et al. [85] synthesized an injectable hydroxyapatite (HAP)-embedded in situ cross-linked hyaluronic acid-alginate (HA-ALG) hydrogel system where exosomes were encapsulated during the in situ gelation step. The nanosized HAP particles were successfully embedded into the HA-ALG hydrogels by high-gravity technology, still maintaining a 3D porous structure. The composite hydrogel allowed continuous and long-term release of hUCMSC-derived exosomes over the course of 14 days where approximately 70% of the loaded exosomes were released after 14 days in vitro. Fan et al. [72] used an injectable MeGC hydrogel to load EMs collected from the noggin-suppressed hMSCs during in situ photo-crosslinking gelation. The MeGC hydrogels incorporated with a high dose of EMs (30 µg) showed more robust bone repair than those with a low dose of EMs (5 µg) in a mouse nonhealing calvarial defect model.

Wang et al. [84] developed a self-healing hydrogel composed of coralline hydroxyapatite (CHA)/SF/glycol chitosan (GCS)/difunctionalized polyethylene glycol (DF-PEG) as a carrier of hMSC-derived exosomes. Exosomes were introduced to a mixture of hydrogel during the step where GCS was dissolved in exosome-containing PBS, and the theoretical loading mass of exosomes per hydrogel was 50 µg. Compared with gelatin hydrogel as control group, CHA/SF/GCS/DF-PEG hydrogels released exosomes more slowly and continuously over the course of 30 days with no initial burst release, demonstrating excellent hydrophilicity of the self-healing hydrogels and their capability as an effective exosome-releasing reservoir. The exosome-integrated hydrogels effectively promoted bone healing in a rat femoral condyle model as evidenced by enhanced BMP2 deposition, collagen deposition and maturation, and angiogenesis.

#### 3.4.3. Surface Modification Strategies

**Polymer coating layer or spheres.** A few studies have introduced supplementary polymer coating layer [63,75,87] or spheres [88] to scaffolds, which enhances the integration stability of exosomes to the scaffolds. Zhai et al. [75] introduced poly-L-lysine (PLL), a positively charged biocompatible polymer, to a 3D porous titanium (Ti) scaffold by incubating the scaffold in a PLL solution overnight. Exosomes derived from hMSCs were then allowed to adsorb to the PLL-coated Ti scaffolds with 200–500 µm-sized pores through electrostatic interactions. The exosome release profiles were monitored for 50 h, but 50% of the loaded exosomes were released only after 2 h, showing an initial burst release phase. Despite the initial burst release, hMSC-exosome coated scaffolds induced enhanced bone tissue regeneration as efficiently as hMSC-seeded exosome-free scaffolds in a rat radial bone defect model after 12 weeks post-implantation.

Diomede et al. [87] exploited polyethyleneimine (PEI), a positively charged biocompatible polymer, to improve the adhesion of hGMSC-derived EVs onto a 3D PLA scaffold. Compared to the control EV group, PEI-EV complexes showed increased internalization to human gingival MSCs in vitro, as cationic PEI favored cellular uptake via proteoglycan binding. Moreover, the 3D PLA scaffolds enriched with PEI-EVs showed more rapid evolution and maturation of new bone nodules and blood vessels in a rat calvarial bone defect model. However, PEI-EV complexes appeared unevenly distributed onto the scaffolds, which needs to be addressed for future applications.

In the work by Li et al. [63], polydopamine (pDA) was introduced to the PLGA scaffolds for strong adhesion and sustained release of hASC-exosomes. Compared with conventional physical adsorption methods, a mussel-inspired pDA coating provided more efficient adhesion of hASC-exosomes onto the PLGA substrates, more than a 2-fold increase in the total mass of exosome loading per scaffold. Without pDA coating, exosomes adsorbed onto the PLGA scaffolds were almost completely depleted within 4 days showing burst release, but exosomes adsorbed onto the pDA/PLGA scaffolds exhibited a slower release of exosomes over the period of 8 days in vitro. Exosome-integrated pDA/PLGA scaffolds successfully promoted bone regeneration in mouse critical-sized calvarial defects.

Swanson et al. [88] loaded exosomes in triblock PLGA-PEG-PLGA microspheres and immobilized those exosome-microspheres to a nanofibrous PLA scaffold for controlled delivery of exosomes. Exosomes derived from human dental pulp stem cells (hDPSCs) were encapsulated in a matrix of PLGA-PEG-PLGA as a product of a water/organic/water emulsion using droplet microfluidics. The exosome-loaded triblock microspheres of uniform sizes were then attached onto the PLA scaffolds using a post solvent-wetting method as shown in Figure 3C. The PLGA-PEG-PLGA spheres effectively protected the encapsulated exosomes, releasing exosomes in a more controlled manner where the release kinetics could be tuned by varying polymer composition. The release rate of exosomes from the microsphere-PLA substrates was nearly constant for up to 10 weeks except the initial burst release (7% in first 24 h) in vitro, demonstrating an increased lifespan of incorporated exosomes. When exosome-microsphere-PLA scaffolds were implanted to mice in vivo, the formation of extracellular matrix was accelerated in subcutaneously implanted constructs, and the craniofacial bone repair was enhanced in calvarial defects [88].

**Streptavidin-Biotin interaction.** The streptavidin (SA)-biotin complex is one of the strongest noncovalent binding partners in nature, offering its versatile use in molecular anchoring systems. Chen et al. [65] utilized the SA-biotin interaction to better immobilize hASC-EVs onto the surface of Ti scaffolds as illustrated in Figure 3D. The Ti surface was first biotinylated using the electrochemical potentiostatic method with polypyrrole (Ppy), a biocompatible and conductive polymer which helps titanium biotinylation. SA was subsequently incubated to form SA-biotin anchors. The membrane of hASC-EVs was biotinylated by adding biotin-labeled lipids, 1,2-distearoyl-sn-glycero-3-phosphoethanolamine-N-[biotinyl(polyethylene glycol)-2000] (DSPE-PEG-Biotin) that embed into the bilayer of hASC-EVs. The biotinylated hASC-EVs were loaded onto the Ti scaffolds having SA-biotin anchors. The hASC-EVs immobilized via SA-biotin binding were able to withstand harsh conditions (ultrasonication to mimic harsh transportation or friction) and survive long-term storage (in PBS at 4 °C for 14 days to simulate a low-temperature transportation). The EV-integrated Ti scaffolds exhibited enhanced bone regeneration ability in the ectopic bone formation mode, which can be ascribed to several osteoinductive miRNA (miR-21, let-7, miR-10a, miR-10b, and miR-199b) encapsulated in hASC-EVs.

**Bioconjugation.** Zha et al. [80] applied a similar strategy to the one in [65] to incorporate exosome-mimetics (EMs) onto the PLA/Chitosan core/shell nanofiber scaffolds. Chitosan on the shell provides amino groups (-NH_2_) that react with the carboxyl groups (-COOH) on biotin through 1-(3-dimethylaminopropyl)-3-ethylcarbonamide hydrochloride (EDC)/n-hydroxysuccinimide (NHS) coupling reactions. After biotin was conjugated with the chitosan shell, SA was introduced for further reaction with biotinylated EMs where parent cells were incubated with DSPE-PEG-Biotin and extruded to generate biotin-modified EMs. In another study, the same group [81] exploited the peptide CP05 as a linker molecule between exosomes shed from a chondrogenic progenitor cell line and the 3D polycaprolactone (PCL) scaffolds. Amino groups were introduced to the 3D-printed porous PCL scaffolds by immersion in 1,6-hexanediamine solution, and the anchor peptides, CP05, were subsequently conjugated to the scaffolds via EDC/NHS coupling reactions. Since CP05 specifically binds to the antigen CD63, a tetraspanin enriched on the exosomal membrane, the CP05-grafted PCL scaffolds showed much higher affinity towards exosomes compared to the control CP05-absent scaffold. Such stable connection between exosomes and CP05-grafted scaffolds led to effective vascularized bone repair in rat segmental bone defects.

## 4. Conclusions and Future Perspectives

In this review, we addressed the liposome- or extracellular vesicle-integrated bone tissue engineering scaffolds where vesicles play a role as bioactive cargo carriers for bone regeneration. Liposomes with tailored design have received great interest as DDS for bone regeneration. Taking advantage of excellent drug loading capability and ease of chemical modifications, liposome-integrated scaffolds have been developed to potentiate their therapeutic potential in bone repair. Efforts have been made to functionalize liposomes to be thermo-responsive, adhesive, bone-targeting, or osteoinductive. However, it should be noted that such functionalization can bring unwanted cytotoxicity of liposomes, e.g., incorporating cationic lipids into liposomes allows loading genetic materials but involves increased cytotoxicity. A careful characterization of potential adverse effects that functionalized liposomes may cause for future clinical applications should be carried out.

EVs have emerged as promising but complex biocompatible DDS for local bone regenerative therapy. Although natural exosomes from various cell lines have been shown to have osteogenic or angiogenic potentials, more systemic and comparative studies need to be examined to leverage the osteogenic potency of EVs. Mechanistic studies on gene expression changes by exosomal miRNA remain to be elucidated. The roles that EVs play as external cargo carriers also need to be further explored in bone regeneration; efficient loading strategies of external cargo (e.g., miRNA) into EVs and the resulting bioactivity of cargo-loaded EVs remain unclear. Because EVs contain numerous surface proteins compared with liposomes, the efficient loading of external cargo into EVs is challenging unlike the case of liposomes. The fine control between increasing cargo loading efficiency and not disturbing the natural lipid and protein composition of EVs should be implemented. The potential synergistic effects to be brought by the delivery of both external cargo and innate exosomal content may promote efficacious bone repair.

We also reviewed the vesicle loading strategies of various biomaterial scaffolds, highlighting recent reports adapting conjugation methods. Conventional loading methods, which confine vesicles relying on physical adsorption, have little impact on local bone repair due to the loss of vesicles accompanying the abrupt release of cargo. The physical adsorption method, due to its simplicity, has been widely applied to load EVs to BTE scaffolds. We draw attention to several strategies employed in liposome-integrated BTE scaffolds that successfully immobilized liposomes packaged with small molecule drugs, suggesting future applications in stably integrating EVs to the scaffolds for bone repair. Given that EVs are also composed of a lipid bilayer membrane with surface proteins sharing structural similarities with liposomes, the liposomal surface modification strategies could be adapted in engineering EVs.

On a final note, measuring release profiles of bioactive molecules in vitro and translating release profiles to in vivo pose technical difficulties. Technological and experimental advances on tracking the kinetic release of vesicles are needed for future clinical applications.

## Figures and Tables

**Table 1:** Summary of cross-linking methods used to integrate liposomes to scaffolds for bone regeneration.

Drug	Liposome Composition	Scaffold	Cross-Linking Method	Notes	Reference
Plasmid DNA encoding RUNX2	DODAP, HSPC, Cholesterol, and DSPE-PEG or DSPE-PEG-Mal	Polycaprolactone nanofiber meshes	Thioether linkage of maleimide and thiol group	Osteogenic activities in human bone-marrow mesenchymal stem cells in vitro	[40]
Dexamethasone	DODAP, HSPC, Cholesterol, and DSPE-PEG or DSPE-PEG-Mal	Polycaprolactone nanofiber meshes	Thioether linkage of maleimide and thiol group	Osteogenic activities in human bone-marrow mesenchymal stem cells in vitro	[44]
Noggin siRNA	Stearylamine and cholesterol	Methacrylated glycol chitosan hydrogel	Encapsulation	Osteogenic and bone regeneration activities in vitro and in vivo	[41]
Phenamil and Noggin siRNA	Stearylamine and cholesterol	Methacrylated glycol chitosan hydrogel	Encapsulation	Osteogenic and bone regeneration activities in vitro and in vivo	[42]
20(s)-hydroxycholesterol and Plasmid DNA encoding sonic hedgehog	Palmitic acid and 20(s)-hydroxycholesterol	Porous hydroxyapatite-coated PLGA scaffold	Electrostatic interaction of alendronate and apatite	Osteogenic and bone regeneration activities in vitro and in vivo	[22]
Kartogenin	Lecithin and cholesterol	Gelatin methacryloyl hydrogel	Encapsulation via the physical network hindrance and non-covalent interaction	Extended joint retention, in vitro chondrogenic activities, and therapeutic effects in osteoarthritis model in vivo	[37]
Dexamethasone	(N-{6-amino-1-[N-(9Z) -octadec9-enylamino] -1-oxohexan-(2S) -2- yl} –N’- {2- [N, N-bis(2-aminoethyl) amino] ethyl} -2-hexadecylpropandiamide) (OO4) and DOPE	Glass coverslips, gold sensors, and silicon substrates	Layer-by-Layer coating with polyethyleneimine, collagen type I, chondroitin sulfate, and liposome	Enhanced adhesion and osteogenic differentiation of C2C12 myoblasts in vitro	[43]
Salvianic acid A	Lecithin, cholesterol, and cholesterol-pyrophosphate	Collagen sponge	Absorption	Improved bone healing via the regulation of HDAC3-mediated endochondral ossification in rabbit segmental defect model	[23]
Deferoxamine and BMP-2	Phosphatidylcholin and Chol	Gelatin methacryloyl hydrogel	Hydrogen bond and hydrogel network micro-cross-linking	Enhanced mechanical property by liposome encapsulation, controlled phase release of various type of drugs, osteogenesis, angiogenesis, mature lamella bone formation in vivo	[38]
Deferoxamine	Lecithin and cholesterol	Gelatin methacryloyl hydrogel and 3D printed bioceramic scaffold	Encapsulation	Designed biomimetic ‘lotus’ biological structure, increased the expression of vascularization, and pro-osteogenic effects in vitro/in vivo	[39]
Curcumin	1,2-dimyristoylsn-glycero-3-phosphocholine (DMPC) and 1,2-dimyristoyl-sn-glycero-3-phospho-(1′-rac-glycerol) (sodium salt) (DMPG)	3D printed calcium phosphate scaffolds	Absorption and ionic interaction	Cytotoxic against in vitro osteosarcoma (bone cancer) cells and promoted osteoblast (healthy bone cell) cell growth	[34]
20(s)-hydroxycholesterol	Stearylamine and 20(s)-hydroxycholesterol	Methacrylated glycol chitosan hydrogel	Encapsulation	Designed non-phospholipid liposome, named sterosome, which has intrinsic osteoinductivity, and enhanced osteogenic activities in vitro and bone formation in vivo via hedgehog signaling	[27]
20(s)-hydroxycholesterol and purmorphamine	Stearylamine and 20(s)-hydroxycholesterol	Porous PLGA scaffold	Polydopamine-mediated layer-by-layer coating (Schiff base formation and Michael-type addition)	Osteogenic activities in vitro and bone formation in vivo via hedgehog signaling	[29]
20(s)-hydroxycholesterol and smoothened agonist (SAG)	Stearylamine and 20(s)-hydroxycholesterol	Porous hydroxyapatite-coated PLGA scaffold	Polydopamine-mediated layer-by-layer coating (Schiff base formation and Michael-type addition)	Osteogenic activities in vitro and bone formation in vivo via hedgehog signaling	[30]
Aspirin and bone forming peptide-1	DSPE-PEG-NH_2_, 1,2- dipalmitoyl-sn-glycero-3-phosphocholine (DPPC), and cholesterol	3D printed polycaprolactone scaffold	Polydopamine-mediated coating	Osteogenic activities in vitro and bone formation in vivo via PI3K/AKT signaling	[36]
Aspirin	DSPE-PEG-NH_2_, DPPC, and cholesterol	3D printed polycaprolactone scaffold	Polydopamine-mediated coating	Osteogenic activities in vitro and ectopic bone formation in vivo	[35]
BMP-2 peptide	HSPC or DPPC, Cholesterol, and mPEG-DSPE-maleimide	Electrospun poly L-lactic acid nanofibers	Thioether linkage of maleimide and thiol group	Sustained release of BMP-2 peptide up to 21 days, enhanced in vitro osteogenic activities, and initiated ectopic bone formation	[33]
107–111 pentapeptide of the parathyroid hormone-related protein (PTHrP 107-111)	MSPC, DSPE-PEG-maleimide, and DPPC	Collagen-hydroxyapatite scaffolds	Thioether linkage of maleimide and thiol group	Triggered release of PTHrP 107-111 by thermal stimulation and enhanced pro-osteogenic activities in vitro	[18]
N′-Dodecanoylisonicotinohydrazide	Phospholipid	PLGA-PEG-PLGA hydrogel	Encapsulation	Developed a liposome-in-hydrogel and sustained drug release	[19]
Carboxyfluorescein, doxorubicin, and lysozyme; not for bone tissue engineering	DSPC, cholesterol, and DSPE-PEG or DSPE-PEG-thiol bisphosphonate	Collagen-hydroxyapatite scaffolds	Electrostatic interaction of bisphosphonate and apatite	Increased affinity to the scaffold and sustained drug release	[21]
BMP-2	Lecithin, cholesterol, and octadecylamine	PEG and Ag ion hydrogel	Encapsulation	Promoted osteogenic differentiation in vitro and local bone remodeling of osteoporotic fracture in vivo because of increased localization efficacy at injected site	[20]
CKIP-1 siRNA	DOTAP, DOPE, cholesterol, DSPE-mPEG2000, and DSPE-PEG2000-maleimide	Bovine bone scaffold	Electrostatic interaction	Osteogenic activities in vitro and bone repair in vivo via CKIP-1 knock down	[25]
